# Association of Sheep Tail Type with the T Gene Single Nucleotide Polymorphisms Loci

**DOI:** 10.3390/life15030342

**Published:** 2025-02-21

**Authors:** Daqing Wang, Yifan Zhao, Wenjing Fang, Junxi Liang, Kuo Li, Caiyun Wang, Guifang Cao

**Affiliations:** 1 College of Veterinary Medicine, Inner Mongolia Agricultural University, Hohhot 010011, China; wangdaqing050789@126.com (D.W.); yifanz311@163.com (Y.Z.); 17684798745@163.com (W.F.); ljx2223128@163.com (J.L.); lkuo777@163.com (K.L.); 2 Animal Embryo and Developmental Engineering Key Laboratory of Higher Education, Institutions of Inner Mongolia Autonomous Region, Hohhot 010011, China; 3 Inner Mongolia Autonomous Region Key Laboratory of Basic Veterinary Medicine, Hohhot 010011, China; 4 College of Life Sciences, Inner Mongolia University, Hohhot 010021, China

**Keywords:** T gene, Hulun Buir short-tailed sheep, Hu sheep, TaqMan probe

## Abstract

This study aimed to develop an effective tail typing detection technology based on the TaqMan probe technology for genotyping different sheep tail types. A total of 122 Hulun Buir short-tailed sheep and 50 Hu sheep were enrolled in the study to compare their tail morphologies, lengths, and widths. Through the Sanger sequencing of loci 333 and 334 in the second exon of the T gene, distinct genotypes of various types of Hulun Buir short-tailed sheep and Hu sheep were identified. In addition, the TaqMan probe technology was employed to genotype the two SNP loci of the T gene in the two types of sheep. It was observed that the second exon of the T gene in Hulun Buir short-tailed sheep at loci 333 and 334 exhibited two genotypes, CT/CT and CT/GG, but this feature was not detected for the T gene in Hu sheep. The detection accuracy of the TaqMan probe technology for sheep tail types exceeded 70%, suggesting that it is an effective tail-typing detection technology. This study provides a solid economic foundation and theoretical ideas that will improve the breeding of short-tailed sheep.

## 1. Introduction

The T gene has been shown to cause variations in animal tails [1], with short-tailed mutant and short-tailed phenotypes collectively referred to as short-tailed malformations. In 1972, Dobrovolskaïa-Zavadskaïa discovered a mutation in the T gene in mice. Notably, mice with the heterozygous genotype exhibited shorter tails compared to those with homozygous genotypes, with some mice showing slight curved tails and others being almost tailless [2,3]. The discovery of homozygous T gene mutations in short-tailed sheep breeds such as Dalagh and Mehraban in Iran, which limit the development of tail vertebrae, also points to a significant role of these mutations in promoting fat accumulation in the tails of large-tailed sheep breeds [4]. The T gene appears to have a dual function, influencing both vertebral growth and fat storage through potential mechanisms involving growth factors, hormonal regulation, and epigenetic factors. This evolutionary adaptation, which allows for the accumulation of fat in the tail, may serve as a survival strategy for periods of food scarcity and as a means to adapt to different environmental conditions. The understanding of this genetic regulation has practical applications in breeding programs for optimizing traits according to agricultural needs, improving nutritional management for livestock, and contributing to comparative genetics research that could extend our knowledge of similar processes in other animals, including humans [5].

Significant research has been conducted on tail type in sheep in the field of animal husbandry [6]. J. Han and Zhida Fu’s study on the genetic basis of the short-tailed phenotype in sheep aimed to unravel the molecular underpinnings of tail development and its influence on production and reproductive processes in sheep. Focusing on breeds with known tail length variation, including the Dalagh and Mehraban breeds, the researchers employed genetic sequencing to analyze the T gene, particularly its second exon, which is crucial for the gene’s functionality. They discovered two significant mutation sites at positions 333 (c.G333C) and 334 (c.G334T) within this exon, which are strongly associated with the short-tailed trait. These mutations, leading to amino acid changes, likely result in a protein product that impacts the development of the tail vertebrae. The study’s findings not only correlate these mutations with the short-tailed phenotype but also suggest that they follow a mendelian pattern of inheritance. The implications of the short-tailed trait on meat production and quality are multifaceted, as it may influence the amount of subcutaneous fat and thus meet quality standards that vary according to market demands. Furthermore, research may have explored the potential effects of tail type on reproductive traits such as fertility and litter size. Understanding the genetic mechanism behind these mutations is key to unraveling the role of the T gene in skeletal development and fat deposition. The practical applications of this research are significant, offering potential improvements in selective breeding practices and the development of genetic tests for identifying carriers of the short-tailed trait. In addition, it provides a new perspective for further research into the genetic mechanisms underpinning sheep tail types to establish strategies for improving sheep breeds [7]. In our laboratory, we conducted whole-genome resequencing of the Hulun Buir short-tailed sheep and utilized sequence analysis for Barag sheep, differing solely in tail morphology [8]. We identified that the T gene mutation (c.G334T) is associated with the short-tail phenotype in Hulun Buir sheep [9]. To validate these findings, we employed the TaqMan probe-based real-time PCR technique, renowned for its sensitivity and specificity. This method facilitates rapid and precise genotyping, crucial for our research. While the TaqMan assay offers high-throughput capabilities, it requires significant investment and technical expertise. Probe and primer design are critical to prevent inaccuracies. The TaqMan probe method for real-time fluorescent quantitative PCR has been applied in diverse fields, such as microbial community characterization, poultry virus infection detection, plant gene identification, aquatic fishery, transgenic detection, and genetic analysis [10,11]. In mutant detection, the TaqMan probe has shown promising results [12]. Two probes with different fluorescent signals were designed, one for detecting the wild type and the other for detecting the mutant, allowing the differentiation of SNP loci based on the different fluorescent signals collected [13]. To detect low-expression genes, mutant genes, and specific gene transcripts, the TaqMan probe method offers greater accuracy and sensitivity compared to the dye method [14]. Considering these advantages, fluorescence quantitative PCR detection using the TaqMan probe method has become one of the most common SNP genotyping methods.

In this study, we employed the TaqMan probes to genotype the T gene SNP loci. A TaqMan probe technique was established for the two mutation sites c.G333 C and c.G334 T in the sheep T gene. Genotyping was performed on the T gene of two SNP loci in Hu sheep and Hulun Buir short-tailed sheep. The results laid a foundation that will guide rapid selection of short-tailed sheep breeding in the future.

## 2. Materials and Methods

### 2.1. Material

A total of 122 Hulun Buir short-tailed sheep (1–2 years old, 61 females and 61 males) were selected from the Xique Breeding Sheep Farm in Hulun Buir, the Aiyiti Livestock Breeding Farm, and the Engineering Experimental Station of the Chinese Academy of Sciences in the Ewenki Banner. Fifty Hu sheep (1–2 years old, twenty fivefemales and twenty fivemales) were obtained from Inner Mongolia Shengle Biotechnology Co., Ltd. Each group of sheep was housed throughout the entire feeding process, with a limited feeding amount. The feeding amount for each experimental sheep was 1.49 kg/d (dry matter intake was 1–3 kg/d). During the experimental period, the feeding environment and management of each group remained consistent, ensuring the cleanliness of the sheep house and sufficient drinking water.

Green Taq Mix, 2×ChamQ Geno-SNP Probe Master Mix (Vazyme, Biotech Co., Ltd., Nanjing, China), DL2000 DNA Marker (TaKaRa Biotechnology Co., Ltd., Dalian, China), blood genomic DNA purification kit, and other reagents and consumables were all purchased from Sangon Biotech Co., Ltd., Shanghai, China.

### 2.2. Observation of Tail Morphology in Hulun Buir Short-Tailed Sheep and Hu Sheep

#### Measurement of Tail Morphology, Length, and Width in Hulun Buir Short-Tailed Sheep and Hu Sheep

The tail lengths and widths of 122 Hulun Buir short-tailed sheep and 50 Hu sheep were measured, and photos were taken to assess the tail morphology of the two types of sheep.

### 2.3. T Gene Sequencing and Tail Type Identification by TaqMan Probe

#### 2.3.1. T Gene Amplification and Identification

Genomic DNA was extracted from blood samples using a blood genomic DNA purification kit. The concentration and A260/280 ratio of the extracted DNA were determined using the NanoDrop 2000 instrument (Thermo Fisher Scientific Co., Ltd., Shanghai, China), with values ranging from 1.8 to 2.0 set as the reference. The genomic DNA samples served as template DNA for subsequent experiments.

According to the T gene sequence (NC_040259) published in the NCBI GenBank data and previous Sanger sequencing results obtained by our research group, the T gene mutation sites were located at positions 333 and 334 of the second exon. Primers were annotated based on the reference sequence, and details of the primers are presented in Table 1.

The PCR amplification was performed using the following reaction protocol: 25 μL Green Taq Mix, 1 μL of DNA template, 2 μL of upstream and downstream primers, and 20 μL of RNase-free ddH2O. PCR was performed under the following thermal cycling conditions: pre-denaturation at 95 °C for 3 min, denaturation at 95 °C for 15 s, annealing at 65 °C for 15 s, extension at 72 °C for 12 s, final extension at 72 °C for 5 min, and a total of 35 cycles. At the end of the reactions, the quality of the PCR products was detected through a 1.0% agarose gel electrophoresis.

#### 2.3.2. TaqMan Probe Genotyping and System Establishment

The probe design was based on the T gene sequence published in the NCBI GenBank database (NC_040259) as the reference sequence. With reference to our previous resequencing results, the T gene mutation sites are located at positions 334 and 333 in the second exon. Based on this information, probes were designed using DNAStar. The “T-HEX” probe was labeled with HEX at the 5′ end and quenched with BHQ1 at the 3′ end. Similarly, the “T-FAM” probe was labeled with FAM at the 5′ end and quenched with BHQ1 at the 3′ end. The primer and probe information are shown in Table 2.

The TaqMan probe reaction system was established using the DNA samples extracted in Section 2.3.1 as the template and the primers and probes are shown in Table 2: 10 μL of 2× ChamQ Geno-SNP Probe Master Mix; 10 μL of DNA template; 1.0 μL of T-HEX and T-FAM; 0.4 μL of forward and reverse primers; and 4.6 μL of RNase-free ddH2O. The reaction mixture was placed in a real-time fluorescent quantitative PCR instrument. The TaqMan probe reaction was performed under the following thermal cycling conditions: pre-denaturation at 95 °C for 3 min, denaturation at 95 °C for 15 s, annealing at 65 °C for 15 s, extension at 72 °C for 12 s, and final extension at 72 °C for 5 min, for a total of 35 cycles. At the end of the reaction, the quality of the PCR products was assessed through 1.0% agarose gel electrophoresis. They were analyzed using the CFX96 software (3.0), which identified three different genotypes.

## 3. Results

### 3.1. Observation Results of Tail Morphology in Hulun Buir Short-Tailed Sheep and Hu Sheep

**Figure 1:**
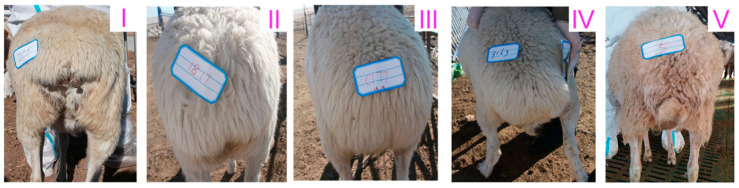
Observation results of tail morphology of Hulun Buir short-tailed sheep and Hu Sheep (**I**). Short-tailed Hulun Buir sheep; (**II**). Medium-tailed Hulun Buir sheep; (**III**). Fat-rumped Hulun Buir sheep; (**IV**). Large-tailed Hulun Buir sheep; (**V**). Hu Sheep.

In the study, we measured tail length and width in 122 Hulun Buir short-tailed sheep and 50 Hu sheep. Those with an average length of 4 cm and an average width of 7 cm were classified as short-tailed Hulun Buir short-tailed sheep (Figure 1I). Those with an average length of 10 cm and an average width of 13 cm were classified as medium-tailed Hulun Buir short-tailed sheep (Figure 1II). Hulun Buir sheep with an average length of 9 cm and an average width of 15 cm were classified as fat-rumped Hulun Buir short-tailed sheep (Figure 1III). Hulun Buir short-tailed sheep were classified as large-tailed if their average tail length was 14 cm and average width was 15 cm (Figure 1IV). Hu sheep exhibited an average tail length of 15 cm and an average width of 8 cm (Figure 1V). 

In summary, the short-tailed Hulun Buir short-tailed sheep had the shortest tail lengths and widths among the Hulun Buir sheep. The Hu had longer tails than all four tail types found in Hulun Buir sheep, but their tail width was comparatively narrower.

### 3.2. Results of T Gene Sequencing and Tail Type Identification by TaqMan Probe

**Figure 2:**
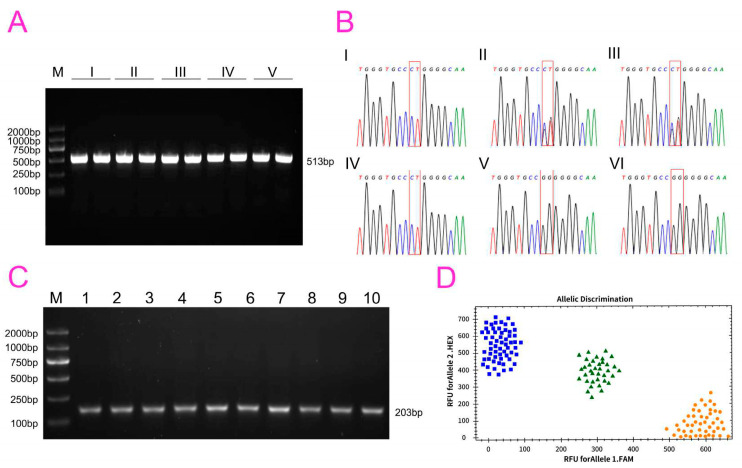
Sequencing of the T gene and identification results of tail types by TaqMan probes. (**A**) Agarose gel electrophoresis of T gene amplification products (Lane M: DL2000; Lane I: T gene amplification product of short-tailed Hulun Buir short-tailed sheep; Lane II: T gene amplification product of fat-rumped Hulun Buir short-tailed sheep; Lane III: T gene amplification product of medium-tailed Hulun Buir short-tailed sheep; Lane IV: T gene amplification product of large-tailed Hulun Buir short-tailed sheep; Lane V: T gene amplification product of Hu sheep.); (**B**); Sanger sequencing results of T gene amplification products ((**I**): T genotype of short-tailed Hulun Buir short-tailed sheep; (**II**): T genotype of short-tailed Hulun Buir short-tailed sheep; (**III**): T genotype of medium-tailed Hulun Buir short-tailed sheep; (**IV**): T genotype of fat-rumped Hulun Buir short-tailed sheep; (**V**): T genotype of large-tailed Hulun Buir short-tailed sheep; (**VI**): T genotype of Hu sheep); (**C**): Verification of primer specificity for TaqMan probe genotyping (Lane M: DL2000; Lane 1–10: PCR products of TaqMan probe genotyping primers); (**D**): Genotyping results of T gene for c.G333C and c.G334T by TaqMan probe method (Blue square: Large-tailed Hulun Buir short-tailed sheep/Hu sheep; Green triangle: Medium-tailed Hulun Buir short-tailed sheep/Short-tailed Hulun Buir short-tailed sheep; Yellow circle: Short-tailed Hulun Buir short-tailed sheep/Fat-rumped Hulun Buir short-tailed sheep).

The samples were subjected to PCR assay followed by 1.0% agarose gel electrophoresis for visualization. All bands could be clearly visualized, and the size of the DNA bands was approximately 500 bp (Figure 2A). The Sanger sequencing results showed that the T genotype of the short-tailed Hulun Buir short-tailed sheep was “CT/CT” (Figure 2B(I)), the T genotype of the short-tailed Hulun Buir short-tailed sheep was “CT/CT” (Figure 2B(II)); the T genotype of the medium-tailed Hulun Buir sheep was “CT/GG” (Figure 2B(III)); the T genotype of the fat-rumped Hulun Buir short-tailed sheep was "CT/CT" (IV); the T genotype of the fat-rumped Hulun Buir sheep and the T genotype of the large-tailed Hulun Buir short-tailed sheep was “GG/GG” (Figure 2B(V)); the T genotype of the Hu sheep was “GG/GG” (Figure 2B(VI)). Following the PCR amplification of the whole genome DNA with the “T SNP–F” and “T SNP–R” primers, the amplicons were resolved through 1.0% agarose gel electrophoresis. The target band ranged between 100 bp and 250 bp, which was consistent with the primer sequences, indicating good primer specificity and suitability for subsequent experiments (Figure 2C). Using the established TaqMan genotyping assay with 10 ng/µL of DNA template, we observed three distinct clusters in the scatter plot: yellow circles, green triangles, and blue squares. Subsequent analysis with CFX96 software (3.0) automatically classified the samples into three corresponding genotypes (Figure 2D). The yellow circular scatter points were delineated using a software as homozygous “CT/CT” at positions 333 and 334 of the second exon of the T gene, which corresponded to short-tailed and fat-rumped sheep.

In this study, we utilized visual inspection and TaqMan probe detection methods to analyze 142 sheep. The TaqMan method identified 40 sheep with large tails, 42 with medium tails, and 38 with short tails. Through visual observation, we identified 52 large-tailed sheep, 60 medium-tailed sheep, and 41 short-tailed sheep. There were 32 Hulun Buir large-tailed sheep and 50 Hu sheep, with 40 of these sheep carrying a GG/GG genotype at positions 333 and 334 of the second exon of the T gene. Further analysis identified that there were 60 Hulun Buir medium-tailed and fat-rumped tailed sheep, with 42 of these sheep carrying a CT/GG genotype at positions 333 and 334 of the second exon of the T gene. Moreover, there were 41 short-tailed Hulun Buir short-tailed sheep, with 38 of these sheep carrying a CT/CT or CT/GG genotype at positions 333 and 334 of the second exon of the T gene. Of note, the TaqMan detection method results were consistent with the Sanger sequencing results.

The coincidence rate between the TaqMan detection and the visual inspection results for large-tailed Hulun Buir short-tailed sheep/Hu sheep was 77%; that for fat-rumped Hulun Buir short-tailed sheep/medium-tailed Hulun Buir short-tailed sheep was 70%; and that for short-tailed Hulun Buir short-tailed sheep was 93%. The details are presented in Table 3.

The above findings indicated that the TaqMan detection could accurately assess the tail-type traits of Hulun Buir short-tailed sheep.

## 4. Discussion

Hulun Buir short-tailed sheep are known for high meat production, low-fat content, high lean meat rate, no mutton odor, and rich nutrition, are thus popular among consumers [15]. This underscores the importance of investigating the genetics of the Hulun Buir short-tailed sheep. To increase the economic benefits of sheep, sheep with shorter tails are often selected for Hulun Buir short-tailed sheep breeding to minimize economic losses caused by offspring with excessively long tails [16,17,18]. This can only be successful if accurate genotyping is performed [19,20,21].

In this study, we measured the tail length and width of 122 Hulun Buir short-tailed sheep (short-tailed type, fat-rumped type, medium-tailed type, and large-tailed type) and 50 Hu sheep (large-tailed type), and found that the tail length of short-tailed Hulun Buir short-tailed sheep was shorter than that of the other three tail types of Hulun Buir short-tailed sheep. Moreover, the tail length of the Hu sheep (large-tailed type) was longer than that of all four tail types of Hulun Buir short-tailed sheep. These findings are consistent with the results reported by Dou Aolei [9], indicating that the average number of tail vertebrae in Hulun Buir short-tailed sheep was lower than that in Hu sheep, and the deformity and shortness of the tail vertebrae in Hulun Buir short-tailed sheep are the key factors causing the short and small appearance of their tail.

The resequencing results of the Hulun Buir short-tailed sheep genome were used to develop the TaqMan probe technology for the two mutation sites c.G333 C and c.G334 T of the sheep T gene, and the two SNP sites (c.G333 C and c.G334 T of the second exon of the T gene) of the T gene in Hulun Buir short-tailed sheep and Hu sheep were genotyped. The obtained results were consistent with those obtained in the Sanger sequencing. The utilization of resequencing data from the Hulun Buir short-tailed sheep genome to develop TaqMan probe technology for the genotyping of two specific mutation sites in the sheep T gene demonstrates the effectiveness of this approach. Notably, this method boasts several advantages over traditional sequencing techniques: it is straightforward to operate, enabling the efficient processing of multiple samples; it is highly sensitive and specific, ensuring the accurate identification of the target mutations; it exhibits good reproducibility, providing consistent results; and it is cost-effective, offering a more affordable option for research. These attributes make the TaqMan (Sangon Biotech Co., Ltd., Shanghai, China) probe technology a valuable tool for genetic analysis, particularly in the context of sheep genotyping and breeding programs, where a rapid, precise, and budget-friendly method is essential [9]. The results indicated that the Hu sheep genotype belonged to the long-tailed breed, while the Hulun Buir short-tailed sheep belong to the short-tailed breed and contained three genotypes at the T gene SNP loci, namely CT/CT, CT/GG, and GG/GG, while the corresponding site of Hu sheep had one genotype, GG/GG.

In further analysis, there was almost no difference in the length of the caudal vertebrae between the large-tailed Hulun Buir short-tailed sheep and Hu sheep, which is consistent with the findings by Dou Aole [9]. J. Han and Zhidafu [8] demonstrated that the short-tailed phenotype in sheep is caused by two mutation sites at positions 333 (c.G333C) and 334 (c.G334T) in the second exon of the T gene. This is in line with the present results. Among the four tail types of Hulun Buir short-tailed sheep, the fat-rumped type exhibits a homozygous mutation at sites 333 and 334 of the second exon of the T gene, specifically CT/CT. The short-tail type carries mutations at these two loci, either CT/CT or CT/GG. In the future, combining these genetic insights with advanced breeding techniques such as genomic selection could significantly enhance the efficiency and precision of the breeding programs for Hulun Buir short-tailed sheep. This may lead to the development of sheep with optimized meat quality, increased resistance to local diseases, and adaptation to specific environmental conditions. Moreover, the economic benefits and simplicity of the genotyping methods used can promote their adoption in both small-scale and large-scale breeding operations, thereby contributing to the sustainable development of the sheep industry. The two loci of 333 and 334 of the second exon of the T gene in the medium-tailed type presented a heterozygous mutation of CT/GG. In comparison, the two loci, 333 and 334 of the second exon of the T gene in the large-tailed type were similar to those of the Hu sheep, both being GG/GG. This indicated that the two loci 333 and 334 of the second exon of the T gene in the large-tailed Hulun Buir short-tailed sheep did not undergo mutation. Evidence from prior investigations indicates that the G mutation to T at the c.G334 T site changes the codon that originally transcribed glutamic acid to one that transcribes tryptophan, which may alter the structure of the Brachyury protein. Mutations within this domain can weaken the transcriptional activation function of the Brachyury protein [22]. The Brachyury protein may affect cell differentiation and proliferation during tail development. For short-tailed sheep, it may promote the differentiation of tail cells to specific types, limit cell proliferation, and cause a short tail to develop. However, in long-tailed sheep, it may act in the opposite way, promoting cell proliferation and specific differentiation, thus forming a long tail.

Subsequently, using the TaqMan probe technology, the five tail-type sheep were genotyped to determine the association between two SNP loci in the T gene and tail traits in Hulun Buir short-tailed sheep and Hu sheep. This led to the identification of c.G333 C and c.G334 T in the second exon of the T gene in sheep, laying a solid foundation for the subsequent breeding and selection of short-tailed sheep.

## 5. Conclusions

In this study, we established a tail-type detection technique using TaqMan probe technology to examine different sheep tail types and genotypic detection. The results showed that the short-tailed Hulun Buir short-tailed sheep exhibited either a CT/CT homozygous mutation or a CT/GG heterozygous mutation. The fat-rumped type carried a CT/CT homozygous mutation, the medium-tailed type demonstrated a CT/GG heterozygous mutation; and both the large-tailed type and Hu sheep exhibited GG/GG without mutations.

## Figures and Tables

**Table 1:** Primer probe information.

Primer Name	Primer Sequence (5′→3′)	Product Length
SNP-F	ACGCGGGGAAGGAAAAGTC	513 bp
SNP-R	CCCCCTCAGCCCCACCTACATC	

**Table 2:** Probe information.

Primer Name	Primer Sequence (5′→3′)	Amplification Product Length	Detection Site
T-HEX	HEX-ACCGAACGGGGGGCCGA-BHQ1		95880385
T-FAM	FAM-CGAACGGGGTCCCGTGGGA-BHQ1		
T SNP-F T SNP-R	TGCGCCCCTTCCTTTTCAG GGGGGAGTCGGGGTGGATGTAG	203 bp	


**Table 3:** The differences in the genotype and typing coincidence rate of sheep with different tail types.

Tail Morphology	Large-Tailed Sheep	Mid-Tailed Sheep	Bob-Tailed Sheep
The tail type contains the flock	Large-tailed Hulun Buir short-tailed sheep/Hu sheep	Fat-rump Hulun Buir short-tailed sheep/medium-tailed Hulun Buir short-tailed sheep	Short-tailed Hulun Buir short-tailed sheep
Genotype detection by Taqman probe (locus 333/334)	GG/GG	CT/GG	CT/CT OR CT/GG
Sanger sequencing genotype (locus 333/334)	GG/GG	CT/GG	CT/CT OR CT/GG
Sequencing Quantity Statistics (Number)	70	42	38
Morphological quantity Statistics (Number)	91	60	41
Anastomosis rate of Taqman probe typing (%)	77 ± 2.4	70 ± 3.2	93 ± 1.8

Data presented as mean SEM.

## Data Availability

Data are contained within the article.
